# Follow-up of phase I trial of adalimumab and rosiglitazone in FSGS: III. Report of the FONT study group

**DOI:** 10.1186/1471-2369-11-2

**Published:** 2010-01-29

**Authors:** Alexandra Peyser, Nathaniel MacHardy, Freya Tarapore, Jacqueline MacHardy, Leslie Powell, Debbie S Gipson, Virginia Savin, Cynthia Pan, Theresa Kump, Suzanne Vento, Howard Trachtman

**Affiliations:** 1 Department of Pediatrics, Division of Nephrology, Schneider Children's Hospital of North Shore-LIJ Health System, 269-01 76th Avenue, New Hyde Park, NY 11040, USA; 2 Unversity of North Carolina Kidney Center, Division of Nephrology and Hypertension, 7012-A Burnett-Womack Building, CB #7155, University of North Carolina at Chapel Hill, Chapel Hill, NC 27599, USA; 3 Department of Medicine, Division of Nephrology, Medical College of Wisconsin, 8701 Watertown Plank Road, Milwaukee, WI 53226, USA; 4 Department of Pediatrics, Division of Nephrology, Children's Hospital of Wisconsin, 999 North 92nd Street, Wauwatosa, WI 53227, USA

## Abstract

**Background:**

Patients with resistant primary focal segmental glomerulosclerosis (FSGS) are at high risk of progression to chronic kidney disease stage V. Antifibrotic agents may slow or halt this process. We present outcomes of follow-up after a Phase I trial of adalimumab and rosiglitazone, antifibrotic drugs tested in the Novel Therapies in Resistant FSGS (FONT) study.

**Methods:**

21 patients -- 12 males and 9 females, age 16.0 ± 7.5 yr, and estimated GFR (GFRe) 121 ± 56 mL/min/1.73 m^2 ^-- received adalimumab (n = 10), 24 mg/m^2 ^every 14 days or rosiglitazone (n = 11), 3 mg/m^2 ^per day for 16 weeks. The change in GFRe per month prior to entry and after completion of the Phase I trial was compared.

**Results:**

19 patients completed the 16-week FONT treatment phase. The observation period pre-FONT was 18.3 ± 10.2 months and 16.1 ± 5.7 months after the study. A similar percentage of patients, 71% and 56%, in the rosiglitazone and adalimumab cohorts, respectively, had stabilization in GFRe, defined as a reduced negative slope of the line plotting GFRe *versus *time without requiring renal replacement therapy after completion of the FONT treatment period (P = 0.63).

**Conclusion:**

Nearly 50% of patients with resistant FSGS who receive novel antifibrotic agents may have a legacy effect with delayed deterioration in kidney function after completion of therapy. Based on this proof-of-concept preliminary study, we recommend long-term follow-up of patients enrolled in clinical trials to ascertain a more comprehensive assessment of the efficacy of experimental treatments.

## Background

Primary focal segmental glomerulosclerosis (FSGS) is increasing in frequency throughout the world [1]. It usually presents with isolated proteinuria or overt nephrotic syndrome in both pediatric and adult patients [2-4]. The cause of this glomerulopathy remains unknown and there are no proven treatments that consistently induce complete remission of proteinuria [5]. Patients who are resistant to corticosteroids and other immunosuppressive medications are at substantial risk of progression to chronic kidney disease (CKD) stage V [6-8]. There is an urgent need to develop new strategies to delay or prevent loss of renal function in this patient cohort.

The primary purpose of the first portion of the Novel Therapies for Resistant FSGS (FONT) study is to evaluate the safety, tolerability, and pharmacokinetic characteristics of novel pharmacological agents that may be antifibrotic and renoprotective. The first two agents selected for testing were rosiglitazone, a peroxisome-proliferator activated receptor-γ, and adalimumab, a human monoclonal antibody to tumor necrosis factor-α. Rosiglitazone is prescribed to children and adults with type 1 and type 2 diabetes [9], while adalimumab is utilized in patients with rheumatoid arthritis and inflammatory bowel disease [10]. The main side effects of rosiglitazone are edema, anemia, congestive heart failure and fractures; the most serious adverse events related to adalimumab use are infections and malignancy [9,10]. In this report, we summarize the kidney function outcomes at follow-up after completion of the Phase I study to obtain preliminary data about the legacy effect of these two drugs, namely their capacity to alter the natural history of the disease in children and young adults with refractory primary FSGS.

## Methods

### Patients

Patients, 2-41 years of age, with biopsy-confirmed primary FSGS and calculated GFR >40 mL/min/1.73 m^2^, were eligible to participate in the FONT study. They were resistant to a standard course of glucocorticoids and had either been treated unsuccessfully with mycophenolate mofetil, azathioprine, cyclosporine, or tacrolimus in the past. The protocol was approved by the Institutional Review Board at each site and patient (and/or parent/guardian) consent was obtained prior to enrollment.

Participants were off all immunosuppressive medications for at least 4 weeks before enrollment. Therapy with angiotensin converting enzyme inhibitors and/or angiotensin receptor blocker drugs was permitted, provided dosages were maintained for the duration of the study. Patients were assigned to receive either rosiglitazone or adalimumab. The total rosiglitazone dose was 3 mg/m^2 ^per day given orally twice a day, with a maximum daily dosage of 8 mg. The adalimumab dose was 24 mg/m^2 ^given as a subcutaneous injection every 14 days, with a maximum single dose of 40 mg. Both experimental agents were given for 16 weeks and patients were evaluated at week 1, 2, 4, 8, 12, and 16. The following clinical and laboratory data were measured at each assessment: blood pressure, height, weight, edema, serum creatinine, estimated GFR (Cockroft-Gault equation if ≥ 18 years and Schwartz formula for < 18 years), urinary protein:creatinine ratio (Up:cr) in a first morning specimen, serum albumin, and blood glucose. In adolescents who passed their 18th birthday during the study, the Schwartz formula was used throughout the observation period. A kidney biopsy at the start or completion of the FONT Treatment Period or at the last follow-up visit was not part of the study protocol. Nineteen out of the 21 patients enrolled in the trial completed the 4-month Treatment Period and laboratory evaluation.

The attending physician of each patient was contacted and asked to provide the patient's serum creatinine concentration and GFRe value for up to 25 months prior to enrollment in the FONT trial. In addition, data about the clinical status and laboratory values at the most recent follow-up assessment were obtained. The treatment after completion of the FONT study was left to the discretion of site nephrologist. In particular, information about current renal status, new medical problems, blood pressure, urinary protein excretion, and GFRe were tabulated. Laboratory tests were performed in local facilities and were not standardized in a central laboratory.

The slope of the line displaying the GFRe over time was calculated for the period prior to enrollment in the FONT Phase I study and for the follow-up period after completion of the 16-week experimental Treatment Period.

### Statistical methods

Data are presented as mean ± SD. Descriptive analyses for demographic variables and laboratories include mean, standard deviation, and median as appropriate. Differences between groups were assessed with a t-test. Results were considered statistically significant if the P value was less than 0.05.

## Results

The clinical status of the 21 patients who participated in the FONT Phase I study at the time of enrollment is summarized in Table 1. The very high GFRe values were obtained in the young children who had correspondingly low serum creatinine concentrations. There were no significant differences between the patients who were assigned to receive adalimumab or rosiglitazone.

**Table 1:** FONT I: Clinical and laboratory features

	*Result*
Age (yr)	16.0 ± 7.5

Pubertal status (<Tanner 3:≥ Tanner 3)	9:12

Gender (M:F)	12:9

Ethnicity (White:Black:Hispanic:Other)	11:5:2:3

Ht (cm)	151 ± 24

Wt (kg)	54 ± 25

Up:cr (mg:mg)	9.3 ± 8.8

GFRe (ml/min/1.73 m^2^)	121 ± 56

The short-term results of the 16-week experimental drug Treatment Period have been described previously [11,12]. Briefly, both agents were generally safe and well tolerated by the patients with resistant FSGS. Two patients were withdrawn from the study before completing the full 16-week Treatment Period - one child assigned to rosiglitazone developed a possible drug allergy (hives and penile swelling), necessitating discontinuation of the drug after 6 weeks and one adult treated with adalimumab was removed from the study after 12 weeks because of refractory edema that required alternate therapy for control. No patient developed significant change in GFRe or was started on renal replacement therapy during the 16-week FONT Treatment Period.

The duration of the follow-up period was similar in the two groups, 18 ± 6 (range: 7-26) and 14 ± 5 (range: 6-20) months in the rosiglitazone and adalimumab groups, respectively (P = 0.12). All of the patients were receiving an angiotensin converting enzyme inhibitor and/or an angiotensin receptor blocker and 10 out of 21 were treated with a statin.

**Figure 1:**
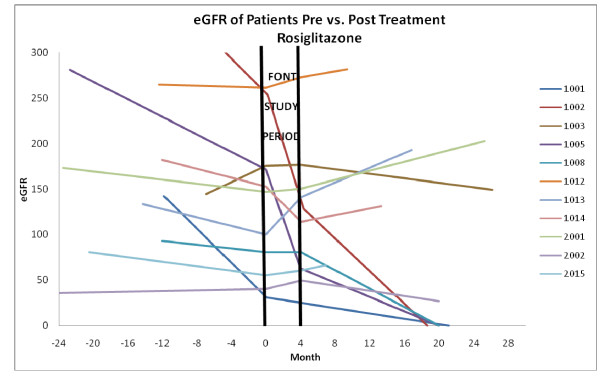
**This graph illustrates the estimated GFR versus time (in months) prior to and after completion of the 6-month FONT Treatment Period in patients assigned to rosiglitazone**.

**Figure 2:**
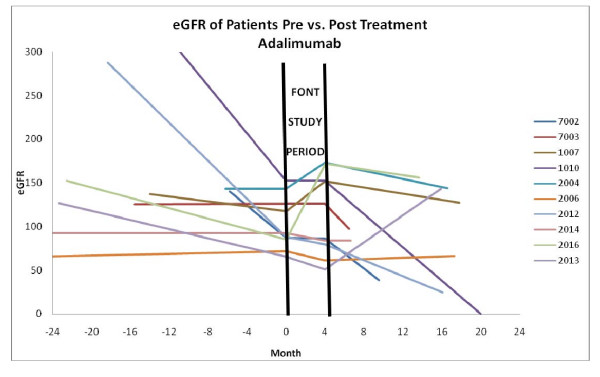
**This graph illustrates the estimated GFR versus time (in months) prior to and after completion of the 6-month FONT Treatment Period in patients assigned to adalimumab**.

During the observation period after completion of the FONT study, 5 patients progressed to CKD stage V - 4 enrolled to the rosiglitazone arm and 1 in the adalimumab group (P = 0.31). Figures 1 and 2 illustrate the linear plots of GFRe *versus *months of observation prior to enrollment in the FONT Phase I trial and after completion of the 16-week experimental Treatment Period for each patient assigned to rosiglitazone (Figure 1) or to adalimumab (Figure 2). The change in the slope of the line relating GFRe *versus *time during the period before and after the FONT study period is summarized in Table 2. There was no significant difference in the change in this value between the group of patients treated with adalimumab compared to those who received rosiglitazone (P = 0.74). The impact of the two FONT therapies on the rate of change in kidney function was not significantly altered if the results of the 5 patients who progressed to CKD Stage V were excluded from the analysis, 1.72 ± 4.10 and 0.49 ± 6.02 ml/min/1.73 m^2^/month in the rosiglitazone and adalimumab groups, respectively (P = 0.65). Overall, among those patients who did not progress to CKD Stage V requiring initiation of renal replacement therapy, a similar percentage in each cohort 5/9(56%) *versus *5/7 (71%) displayed stabilization in GFRe therapy during the observation period after completion of the FONT study, based on a less steep slope of the GFRe *versus *time line (P = 0.68).

**Table 2:** Change in GFRe in response to FONT study intervention: Rosiglitazone *versus *adalimumab

Patient #	Rosiglitazone			Adalimumab		
	**Follow-up interval (months)**		ΔGFRe slope*	**Follow-up interval (months)**		ΔGFRe slope*

	**Pre**	**Post**		**Pre**	**Post**	

1	12	21	7.91	6	10	-0.39

2	25	19	0.46	16	6	4.04

3	7	26	-5.73	14	18	-2.28

4	23	20	0.91	11	20	0.12

5	12	20	-4.01	50	17	6.35

6	12	9	1.96	6	17	10.36

7	14	17	6.42	23	16	0

8	12	13	4.23	18	16	1.33

9	24	25	3.58	32	7	0.62

10	25	20	-1.65	23	14	-11.69

11	20	7	3.23			

Mean ± SD	16.8 ± 6.4	18.0 ± 6.0	1.72 ± 4.10	20 ± 13	14 ± 5	0.84 ± 5.78

*The change in slope is reported as ml/min/1.73 m^2^/month

## Discussion

There is an urgent need to systematically develop and evaluate novel therapies for patients with resistant primary FSGS because the disease is becoming more prevalent and the risk of progressive loss of kidney function is especially high in those who do not achieve a reduction in proteinuria in response to standard immunosuppressive therapy. The rationale for the FONT trial is that absent a consistent immunolopathological target of treatment, interventions designed to reduce renal fibrosis offer the best hope of stabilizing kidney function and preventing or attenuating the steady decline in kidney function and the need for renal replacement therapy. This approach is feasible in patients with primary FSGS, irrespective of whether it is linked to genetic mutations in podocyte proteins or if there is no demonstrable molecular basis for the glomerular disease [13].

The results of FONT Phase I study indicate that adalimumab and rosiglitazone are safe and generally well tolerated in patients with primary FSGS. In addition, pharmacokinetic analyses indicate that because of a reduced area under the curve and enhanced clearance, the dosage of both agents needs to be modified upward in order to achieve drug levels that are comparable to those observed when the drugs are used for FDA approved indications in patients with normal kidney function and without nephrotic-range proteinuria [11,12]. The outcomes of the two Phase I trials justify moving forward with the assessment of these two agents. Clarification of any benefit of rosiglitazone and/or adalimumab therapy requires the performance of a randomized clinical trial in which the efficacy of these antifibrotic agents is compared to a parallel group given conservative medical therapy alone - an angiotensin converting enzyme inhibitor, an angiotensin receptor blocker, and a statin. This question hopefully will be answered in the ongoing FONT Phase II trial.

Determination of the efficacy of adalimumab and rosiglitazone as antifibrotic agents in resistant primary FSGS will require performance of Phase II and III randomized controlled studies that enroll a sufficient number of patients and have statistical power to demonstrate a clinically significant effect on hard endpoints such as change in GFRe or on surrogate markers like proteinuria. However, therapeutic treatment period and follow-up interval in clinical trials are often not long enough to adequately demonstrate differences in clinically relevant hard renal outcomes such as doubling of serum creatinine and need to initiate renal replacement therapy [14]. Therefore, an important consideration in evaluating novel therapies for renal disease is to ascertain whether the impact of the experimental intervention is prolonged and is manifest even after discontinuation of the study drug. This accounts for our focus in this report on GFRe rather than surrogate markers like proteinuria and blood pressure that are detailed in the primary reports of the FONT Phase I trials [11,12]. The FDA has strongly recommended that this type of assessment be incorporated into the analysis of efficacy novel therapies for glomerular disorders [15]. The 'legacy" effect has been examined in studies that tested interventions to lower serum glucose concentration and blood pressure in patients with diabetes [16-18]. However, this type of analysis has not become a standard element in the design of clinical trials in nephrology, especially those involving pediatric patients.

This report represents an initial step in this direction by comparing the slope of the GFRe versus time curve prior to and after treatment with adalimumab or rosiglitazone in the FONT Phase I study. To the best of our knowledge, this is first time such an assessment has been conducted in patients with primary FSGS. The results indicate that approximately 30% of 21 patients had a clinically measurable slowing of the rate of deterioration in kidney function for up to a year after the 16-week treatment with either adalimumab or rosiglitazone. We acknowledge that the number of patients in each group is small, consistent with a Phase I study. Therefore, it is premature to draw any conclusion about the efficacy of the test therapies without studies involving a larger cohort given the antifibrotic agents for an extended period or to make any meaningful comparison between the two treatments.

There are several limitations to this study. The use of formulas to estimate GFR in children and adults is the subject of ongoing controversy. Newer formulas have been proposed that may predict the level of kidney function more accurately [19,20]. The age-appropriate Schwartz and Cockcroft-Gault formulas were used consistently for the entire study period and, therefore, changes in GFRe should not be effected by shortcomings in the formulas, per se. We did not rely on the MDRD formula which is inaccurate in patients with GFRe greater than 60 ml/min/1.73 m2 [21]. In addition, the serum creatinine measurements used to calculate GFRe during follow-up were not performed in a central laboratory or standardized. Nonetheless, they were consistent for each patient and should reflect disease progression in individual cases.

We acknowledge that it would be premature to speculate on the frequency and magnitude of long-term benefit of antifibrotic therapy based on this small relatively heterogeneous cohort. The clinical ramifications of the change in the slope of the GFRe versus time line needs clarification in larger series of patients. However, one cannot gainsay the value of studies like the FONT trial in which there are well defined criteria for enrollment and patient management is controlled. Finally, because the follow-up was conducted after completion of the formal protocol, the treatments that the patients in each group received were uncontrolled. Therefore, we cannot attribute any beneficial long-term effect on GFRe to a delayed effect of the FONT antifibrotic interventions or to the uncontrolled therapies prescribed during the post-FONT treatment period.

## Conclusion

The information in this preliminary report supplements our projected effect of these drugs in a Phase II trial, in which we have calculated the sample size based on the occurrence of a 50% reduction in proteinuria in 30% of patients are treated with adalimumab or rosiglitazone *versus *10% of patients who are given conservative medical therapy (angiotensin converting enzyme inhibitor, angiotensin receptor blocker, and HMG-CoA reductase inhibitor). The findings of long-term follow-up from the FONT Phase I studies need to be extended and confirmed in larger Phase II and III trials. However, they suggest that the use of antifibrotic agents for a defined period of time may have a legacy effect and represent a viable strategy to preserve kidney function in glomerular disorders like FSGS that are resistant to corticosteroids and other immunosuppressive drugs. Based on this preliminary proof-of-concept study, we recommend that long-term follow-up be incorporated into the study design of all clinical trials in nephrology that include pediatric and/or adult patients in order to enable more comprehensive assessment of the effect of the experimental treatments under evaluation (word count: 2301).

## Abbreviations

CKD: Chronic kidney disease; FONT: Novel therapies for resistant FSGS Study; FSGS: Focal segmental glomerulosclerosis; GFRe: estimated glomerular filtration rate; TNF: Tumor necrosis factor.

## Competing interests

The authors declare that they have no competing interests.

## Authors' contributions

AP organized and analyzed the follow-up data and prepared the graphs. NH and FT organized and analyzed the follow-up data. JH, LP, TK, and SV collected the follow-up information from the patients. VS and CP participated in the study design. DG and HT conceived the study design, supervised the project, interpreted the data, and drafted the manuscript. All of the authors read and approved the manuscript

## Pre-publication history

The pre-publication history for this paper can be accessed here:

http://www.biomedcentral.com/1471-2369/11/2/prepub
